# Implications of sequence variation on the evolution of rRNA

**DOI:** 10.1007/s10577-018-09602-w

**Published:** 2019-02-05

**Authors:** Matthew M. Parks, Chad M. Kurylo, Jake E. Batchelder, C. Theresa Vincent, Scott C. Blanchard

**Affiliations:** 1 Department of Physiology and Biophysics, Weill Cornell Medicine, New York, NY USA; 2 Department of Immunology, Weill Cornell Medicine, New York, NY USA; 3 Department of Immunology, Genetics and Pathology, Uppsala University, Uppsala, Sweden; 4 Tri-Institutional PhD Program in Chemical Biology, Weill Cornell Medicine, New York, NY USA

**Keywords:** concerted evolution, ribosomal RNA, protein synthesis, specialized ribosomes, rDNA, rRNA, genome evolution

## Abstract

The evolution of the multi-copy family of ribosomal RNA (rRNA) genes is unique in regard to its genetics and genome evolution. Paradoxically, rRNA genes are highly homogenized within and between individuals, yet they are globally distinct between species. Here, we discuss the implications for models of rRNA gene evolution in light of our recent discoveries that ribosomes bearing rRNA sequence variants can affect gene expression and physiology and that intra-individual rRNA alleles exhibit both context- and tissue-specific expression.

The ribosome is an RNA-protein assembly responsible for the translation of the genetic information encoded in messenger RNA (mRNA) into proteins. The ribosomal RNA (rRNA) component of the intact ribosome, which constitutes its universally conserved, functional core (Noller 2005), is principally encoded by the ribosomal DNA (rDNA) operon (Parks et al. 2018; Grummt 2003). Most eukaryotic genomes encode hundreds of copies of the rDNA operon arranged in tandem repeats on the acrocentric arms of multiple chromosomes (Eickbush and Eickbush 2007). These operons encode the functional rRNAs found in the fully assembled ribosome as well as non-coding RNAs of potentially diverse physiological functions (Bierhoff et al. 2014).

To meet cellular demands for rapid protein synthesis during cellular growth and proliferation, rDNA operons are highly transcribed by RNA Polymerase I. Pre-rRNA transcripts (47S in higher eukaryotic organisms) are processed into the functional rRNAs (5.8S, 18S, and 28S) present within the ribosome through a highly complex multi-step process referred to as ribosome biogenesis (Peña et al. 2017). In addition to its interactions with hundreds of ribosome biogenesis factors, the rRNA components of the assembled ribosome must precisely interact with more than 80 ribosomal proteins and a multitude of translation factors and other components within the cellular milieu (Simsek et al. 2017). Correspondingly, rRNA is thought to be highly intolerant to sequence variation within a given species, thus requiring the homogenization of allelic variation to maintain a diversity of structure-function relationships. Despite this constraint, both the sequence and structure of rRNAs have been remodeled, sometimes dramatically, over evolutionary time (Petrov et al. 2014; Bernier et al. 2018). The physiological impacts of rRNA sequence variation during the evolution of rRNA genes have thus been proven to be a special case with respect to conventional notions of evolution.

Two accepted scientific observations give rise to an apparent paradox in rRNA evolution: on the one hand, rRNA genes are highly similar within and between individuals of a given species (Eickbush and Eickbush 2007). On the other hand, they are sufficiently divergent to distinguish one species from another using rRNA genes alone, including closely related primates (Arnheim et al. 1980). How then can the hundreds of rRNA genes be maintained as highly homologous across individuals and generations, while significantly diverging to a new consensus sequence during the emergence of a new species? If rRNA genes are homogenized, then the appearance of a new consensus rRNA sequence with the emergence of a new species must have arisen from a de novo mutation in one gene, which then quickly spread to become ubiquitous to all other copies of rRNA across non-homologous chromosomes, leaving little room for selection in the interim (Fig. 1). To be inheritable, these events must occur in meiotic cell lineages.

**Fig. 1:**
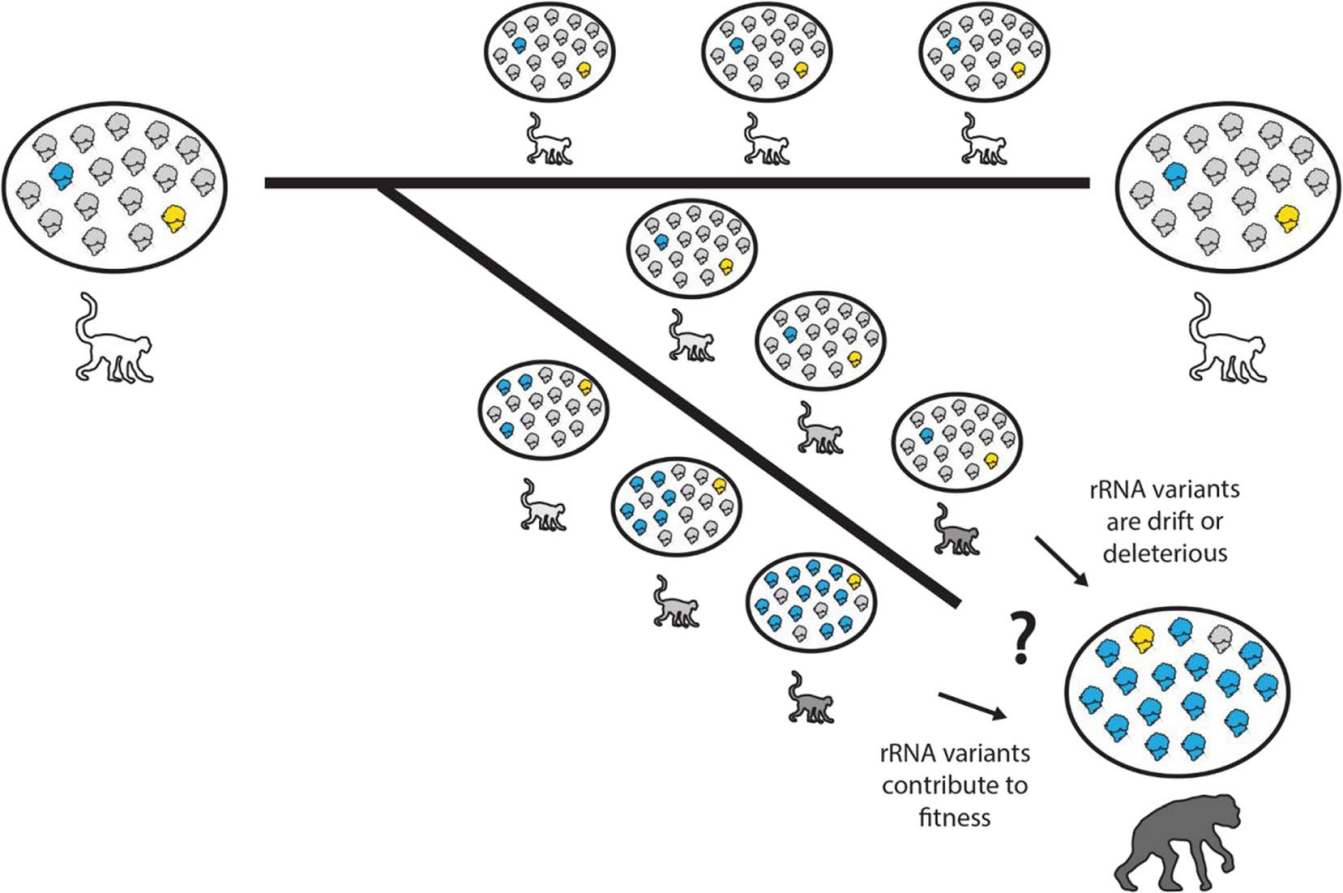
How do the hundreds of copies of rRNA genes evolve during speciation? Schematic model of a speciation event. Cells (black circles) containing translating ribosomes bearing different rRNA alleles (gray, blue, or yellow) correspond to representative individuals (white or shaded primate outlines) depicted during speciation. In a model of concerted evolution, wherein rRNA variants are only deleterious or genetic drift, the rRNA genes are maintained homologous while the species evolves, but the consensus rRNA gene in the emergent new species differs from the ancestor due to some sudden meiotic recombination event, which spreads the variant to all copies of the rRNA gene in the genome. Here, we propose an update to the model of concerted evolution, wherein rRNA variants can contribute beneficially to cell physiology and certain variants can be selected for as the species evolves

Concerted evolution, a genetics model proposed in the wake of these observations, seeks to provide a framework for rRNA evolution that entails a combination of gene conversion and unequal chromosome crossover (Eickbush and Eickbush 2007), mechanisms of recombination, and DNA repair that induce genome rearrangements among repetitive DNA (Parks et al. 2015; Hastings et al. 2009). An assumption underlying concerted evolution is that the precise nucleotide sequence of rRNA must be maintained for proper functioning of the ribosome (Eickbush and Eickbush 2007; Brown et al. 1972). In its present form, concerted evolution regards rRNA sequence variants as inconsequential genetic drift or deleterious mutations (Eickbush and Eickbush 2007; Brown et al. 1972).

Mounting evidence hints that the evolution of rRNA sequence variation is more complicated than the rapid spread of mutations across rRNA genes and the simple avoidance of deleterious mutations. In 1980, Arnheim et al. reported an evolutionarily conserved intra-individual 28S rRNA sequence polymorphism in four primate species in which the same nucleotide polymorphism was present in the 28S rRNA gene of only a subset of each individual’s rRNA genes (Arnheim et al. 1980). In human samples, this sequence polymorphism was estimated to be present in 30% of the genes (Krystal et al. 1981). The authors noted that concerted evolution could not explain the existence of such a conserved intra-individual polymorphism and speculated that a selection pressure prevented removal of this variant through homogenization. Subsequent discoveries of intra-individual nucleotide polymorphisms in human rRNA genes led Gonzalez et al. to speculate that rRNA variants may regulate gene expression, such that small genotypic differences in rRNA genes may have substantial phenotypic consequences (Gonzalez et al. 1985). As rRNA gene clusters had been evidenced to exhibit tissue- and condition-specific expression (de Capoa et al. 1985a; de Capoa et al. 1985b), the ramifications of such findings appeared profound, albeit difficult to explore.

Consistent with the notion of concerted evolution, we recently performed a focused analysis on the functional rRNA genes using relatively short-read length human whole genome sequencing data from diverse populations worldwide to show that mammalian rRNA genes are, in general, highly homologous (Parks et al. 2018). However, we also discovered intra-individual sequence variation across the rRNA genes, some of which occurred in regions of functional relevance and were associated with population groups. rRNA variants were also found to be conserved between human and mouse and to exhibit tissue-specific expression. These discoveries raise the possibility that sequence variation in the rRNA component of the ribosome may be of beneficial functional significance. These observations led us to ask whether the expression of rRNA sequence variants can affect gene expression and phenotype.

Based on the rationale that beneficial impacts of sequence variation within the rRNA genes on ribosome function, if present, would likely be an evolutionarily conserved phenomenon, we examined whether rRNA sequence variation contributes to physiological programs in laboratory strains of *Escherichia coli*. We discovered that rRNA alleles are differentially expressed in response to nutrient limitation-induced stress and that the rRNA allele most upregulated on a relative basis is distinguished by conserved sequence variants clustered in the small subunit head domain of the assembled ribosome (Kurylo et al. 2018). These findings revealed for the first time that the rRNA allele composition of the actively translating ribosome pool is indeed regulated in response to physiological stimuli. Remarkably, we further showed that ribosomes bearing these sequence variations causally affected stress-response gene expression and phenotype, including biofilm formation, cell motility, and antibiotic sensitivity. Consistent with the sequence variants modulating the so-called ribo-interactome (Simsek et al. 2017), we further identified rRNA allele-dependent genetic interactions with stress-related proteins that transiently interact with the ribosome in the proximity of the sequence variants during protein synthesis. The varied expression of this operon alters the mechanisms of ribosome-interacting factor engagement during translation to affect gene expression and cell physiology during stress. Interestingly, the exact same sequence variations were found to be conserved in many *Enterobacteriaceae*, including *Salmonella enterica*, which diverged from *E. coli* more than 120 million years ago. These findings provide compelling evidence that classes of bacteria encode what may be considered an evolutionarily conserved “stress-response ribosome.” As the theory of concerted evolution has been extended to prokaryotes with multiple copies of rRNA (Liao 2000), these results point towards a new dimension of rRNA gene evolution. We note, however, that context-specific benefits for the expression of rRNA genes with sequence variation within pre-rRNA or the rRNA genes have yet to be definitively shown in eukaryotes.

In light of previous literature and the more recent discoveries described herein, we posit that concerted evolution should be expanded to account for the possibility that positive selective pressures favor the maintenance of a plurality of rRNA alleles in the genome (Fig. 1). This may include pressures that favorably modulate ribosome function in certain environmental conditions or cellular states. For instance, the rRNA sequence variants that we observed to be conserved in *Enterobacteriaceae* appear to modulate ribosome function in a manner that influences gene expression and cell physiology to promote survival. Analogously, extremophilic or extremotolerant microorganisms seem to possess unusually high levels of intragenomic rRNA sequence variation (Sun et al. 2013), possibly contributing to their ability to navigate harsh environmental conditions (Lauro et al. 2007; López-López et al. 2007; Johansen et al. 2017).

In addition to uncovering a novel mechanism of gene expression regulation, our recent findings raise important questions about concerted evolution and rRNA. For instance, how do sequence variants in rDNA operons arise in the first place and how are they maintained? Do sequence variations within the rRNA genes correlate with variations within the non-coding regions of the pre-rRNA transcript? How do the potentially distinct constraints on sequence variation within the genes encoding the functional rRNA genes and the non-coding regions of rDNA operon influence and impact the mechanism and occurrence of rDNA recombination events? How is an optimal plurality of rRNA alleles achieved within an individual and is this plurality maintained over the course of an organism’s lifetime? Do the positions of the rDNA operons within the acrocentric arms of chromosomes alter the efficiency of recombination events in a meaningful way? We must also seek to understand how ribosome biogenesis and translation factors coevolve to maintain functional interactions with rRNAs of distinct sequence. Do variations in rRNA sequence drive the evolution of ribosome biogenesis and translation factors or do variations in ribosome biogenesis and translation factors drive the evolution of rRNA sequence? How does rRNA sequence variation relate to the observation that mammalian organisms typically encode multiple distinct isoforms of the core translation factors (Genuth and Barna 2018)?

To begin to answer these questions and to provide insights into the role of rRNA expression in concerted evolution, future investigations must be aimed at examining whether diverse eukaryotic species exhibit rRNA allele-specific expression levels under distinct physiological conditions. In this context, efforts must also be given to establishing a robust means of determining the complete primary sequence of individual rDNA operons within a single cell or organism. As it is difficult to achieve this goal with short-read length whole genome sequencing data, such efforts will doubtless benefit from the implementation of single-chromosome or single-molecule, long-read length technologies (Pollard et al. 2018). Equipped with this knowledge, one could endeavor to genetically or pharmacologically regulate the levels at which specific variant rDNA operons are expressed. In doing so, one could assess the impact of ribosomes composed of certain rRNA alleles on gene expression and phenotype and track rRNA genotypes over time. Such knowledge will inform targeted knock-in and knock-out studies to solidify the contribution of rRNA sequence variation in gene expression and physiology. It will also provide a much-needed foundation for genome-wide association studies, which may identify rRNA alleles to be implicated in disease conditions. These important first steps may be critical to motivating the necessary tool developments, genomics approaches, and research collaborations needed to navigate and chart this important research frontier.

As we touch upon herein, rRNA may be subject to distinct evolutionary processes and pressures. Understanding how rRNA alleles are maintained in the genome and regulated in expression will be of extraordinary importance given the centrality of the ribosome to cellular physiology. In light of the evidence showing that rRNA sequence variation has the capacity to regulate gene expression and cell physiology (Kurylo et al. 2018), as well as evidence showing a connection between genomic instability of rDNA and disease (Stults et al. 2009), these areas of investigation appear both critical to pursue and distinctly uncharted.

## Abbreviations

mRNA: Messenger RNA
rDNA: Ribosomal DNA
rRNA: Ribosomal RNA
