# Correction to: Implications of sequence variation on the evolution of rRNA

**DOI:** 10.1007/s10577-019-09610-4

**Published:** 2019-06-28

**Authors:** Matthew M. Parks, Chad M. Kurylo, Jake E. Batchelder, C. Theresa Vincent, Scott C. Blanchard

**Affiliations:** 1 Department of Physiology and Biophysics, Weill Cornell Medicine, New York, NY USA; 2 Department of Immunology, Weill Cornell Medicine, New York, NY USA; 3 Department of Immunology, Genetics and Pathology, Uppsala University, Uppsala, Sweden; 4 Tri-Institutional PhD Program in Chemical Biology, Weill Cornell Medicine, New York, NY USA

**Correction to: Chromosome Res (2019) 27:89–93**

10.1007/s10577-018-09602-w

The article Implications of sequence variation on the evolution of rRNA, written by Matthew M. Parks, Chad M. Kurylo, Jake E. Batchelder, C. Theresa Vincent and Scott C. Blanchard, was originally published electronically on the publisher’s internet portal (currently SpringerLink) on February 05, 2019 without open access.

With the author(s)’ decision to opt for Open Choice the copyright of the article changed on May 2019 to © The Author(s) 2019 and the article is forthwith distributed under the terms of the Creative Commons Attribution 4.0 International License (http://creativecommons.org/licenses/by/4.0/), which permits use, duplication, adaptation, distribution and reproduction in any medium or format, as long as you give appropriate credit to the original author(s) and the source, provide a link to the Creative Commons license and indicate if changes were made.

The original article has been corrected.

